# Quality and Safety of Rural Community Drinking Water Sources in Guto Gida District, Oromia, Ethiopia

**DOI:** 10.1155/2021/5568375

**Published:** 2021-05-25

**Authors:** Motuma Tessema Abegaz, Mulissa Jida Midekssa

**Affiliations:** ^1^Department of Biology, College of Natural and Computational Sciences, Wollega University, Nekemte, Ethiopia; ^2^Environmental Biotechnology Directorate, Ethiopian Biotechnology Institute, Addis Ababa, Ethiopia

## Abstract

The quality of drinking water has always been a major public health concern, especially in developing countries where access to improved water supply and sanitation is very low. This study aimed to assess the bacteriological and physicochemical quality of rural community drinking water sources in the Guto Gida district. A cross-sectional study was conducted in selected rural areas of the district from January to June 2016. Water samples were collected from four types of sources (protected dug well, open dug well, protected spring, and open spring) found in 8 locations of the study area. The membrane filtration technique was employed to determine the total coliform and faecal coliform load of the samples. The physicochemical characteristics such as total dissolved solid (TDS), pH, electrical conductivity (EC), turbidity, temperature, color, iron, manganese, lead, fluoride, zinc, sulphate, nitrate, and phosphate were analyzed following the American Public Health Association and WHO standard protocols. Our results revealed that 90.6% and 87.5% of water samples were positive for total coliform and faecal coliform, respectively. Thus, the majority of the studied water sources could be classified as polluted with respect to coliform load. Our results also have shown that most of the water sources showed marginally tolerable quality with respect to color, EC, TDS, turbidity, nitrate, sulphate, and phosphate. However, the protected sources had poor quality in zinc, lead, iron, manganese, and pH with values above the permissible levels. Thus, the drinking water source quality of the study areas requires appropriate interventions such as improving the existing water source infrastructure and access to sanitation services.

## 1. Introduction

Water is the most abundant compound, which plays a significant role in maintaining the health and welfare of human beings. Nevertheless, its quality and suitability for use are determined by its taste, odor, color, and the concentration of organic and inorganic matter found in it [1]. The quality of drinking water can be compromised when it is contaminated by waste from various sources. The sources of water contamination could be geological, industrial, and agricultural activities. These contaminants are further categorized as microorganisms, inorganics, organics, and radionuclides. They can affect the quality of water and then human health upon consumption before proper treatment.

Safe and adequate water supply is a vital element to preserve human health and hence access to clean drinking water is now recognized as a fundamental right of human beings. Achieving universal access to safe drinking water and sanitation services is a priority in global development policy as promulgated in Goal 6 of the Sustainable Development Goals (SDGs). Nevertheless, access to clean water is still limited in many developing countries. Hence, more than 700 million people, mostly living in developing countries, have no access to improved water sources and sanitation facilities [2]. Lack of adequate sanitation services could cause water contamination and lead to a number of diseases such as cholera, dysentery, salmonellosis, and typhoid [3]. Waterborne diseases associated with these are attributed to the death of millions in developing countries every year.

Though Ethiopia has met the 2015 Millennium Development target of providing drinking water from improved sources [4], the country is among the lowest in sub-Saharan countries in terms of rate of access to safe drinking water [5]. Despite all the efforts of UNICEF and the Water, Sanitation, and Hygiene (WASH) project on well and spring water development and latrine construction activities, the problem of water quality is still rampant in the rural part of Ethiopia. Globally, the gap in the provision of piped drinking water between urban and rural communities is highly pronounced [4]. Likewise, in Ethiopia, only 57% of households have access to an improved drinking water source, with a higher (93%) proportion among urban residents and a lower (49%) proportion among rural residents [6]. This clearly indicates that there is a wide disparity between urban and rural communities with respect to safe drinking water supply coverage. Hence, the majority of rural communities in the country have no access to piped water and improved water sources. Consequently, their primary water sources are mostly developed springs and hand-dug wells, shallow and deep-drilled wells, and ponds. Moreover, unimproved sanitation habits and open defecation practices commonly observed among rural communities of the country have exacerbated the problem of water quality [7].

According to WHO/UNICEF [4], improved drinking water sources are defined by the nature of their design and construction, have the potential to deliver safe water, and include piped water, boreholes or tube wells, protected dug wells, protected springs, rainwater, and packaged or delivered water. In Ethiopia, most of the population relies on water sources that are unimproved, such as ponds, lakes, rivers, and open dug wells. There are sources of drinking water that are poorly constructed or do not have any engineered facilities such as spring box, borehole capping, and wells [8]. On the other hand, several previous studies conducted in Ethiopia showed that rural community drinking water sources were commonly contaminated with indicator bacteria and other pollutants associated with poor water supply and sanitation [4, 9–15]. Microbial contamination is among the most common health risks associated with drinking water [5]. According to both the WHO microbiological guidelines [16] and Ethiopian drinking water quality standards [17], coliform bacteria should not be detected in 100 ml samples of water for the water to be considered safe; their detection in water indicates pathogenic bacterial contamination [5, 18].

In most rural areas, animal waste, garbage, and liquid waste are commonly disposed of inappropriately in the surrounding fields [7, 19]. These situations may lead to contamination of water sources [2]. Water can be contaminated at any point in the supply system [19]. Although protection of water supply from contamination is the first line of defense, water source protection is the best method of ensuring safe drinking water [12]. Thus, a continuous surveillance and monitoring system of water sources should be in place to ensure the provision of safe and good quality drinking water for rural communities [3]. The provision of safe and adequate water supply for the population has far-reaching effects on health, productivity, and quality of life [20]. Therefore, this study was designed to assess the bacteriological and physicochemical quality of rural community drinking water sources from selected sites in the Guto Gida district.

## 2. Methodology

### 2.1. Description of the Study Site

**Figure 1:**
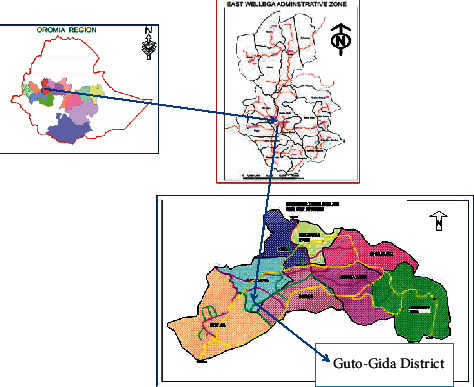
Map of the study area.

This study was conducted in the Guto Gida woreda, which is one of the districts of the East Wollega Zone, Oromia (Figure 1). The district is divided into 21 rural *Kebeles* (the smallest local administrative unit) and one town. The total number of population in the district is 105,332 in 2005 E.C., out of which 97.22% live in rural areas and are directly engaged in agriculture. About 70% of households in these areas are dependent on 209 wells (14 protected wells and 195 unprotected wells) and 49 springs (11 protected and 38 unprotected) as a source of water, whereas 30% of the households are using tap water as a source of drinking water.

### 2.2. Study Design and Sample Collection

A cross-sectional study was conducted in rural areas of Guto Gida woreda (district) from January to June 2016 to assess the bacteriological and physicochemical quality of drinking water sources. The study sites were selected from the Guto Gida district using a simple random sampling system. However, sources at the site were selected purposely to include protected dug well, open dug well, protected spring, and open spring water sources in the same sites based on the number of peoples depending on the sources. Accordingly, a total of 32 triplicate water samples were collected from the four types of water sources found in eight locations of the study area. Water samples were collected into sterile plastic bottles as described in the WHO guidelines [21] and transported to Nekemte Water and Sewerage Service Enterprise Laboratory in iceboxes. Bacteriological analysis was done within 3-4 hours of collection.

All water sampling and preservation procedures were performed according to the standard methods, American Public Health Association for the Examination of Water and Wastewater [22], and WHO guidelines for drinking water [16]. Sampling of water for bacteriological analysis was done aseptically with care, ensuring no external contamination of samples. Water samples were collected by direct flow into sterilized bottles, sealed, and placed in an insulated box to keep the temperature below 4°C. Dug well water samples were collected using containers used by the communities and then transferred into sterile bottles. Temperature, pH, EC, and TDS were measured at the sites of collection with portable equipment following standard protocols. Samples were transported to the laboratory in iceboxes and analyzed immediately.

### 2.3. Analysis Bacteriological and Physicochemical Characteristics of the Water Sources

#### 2.3.1. Physicochemical Analysis of the Water Sources

The physicochemical analysis carried out in this study included temperature, turbidity, EC, TDS, color, iron, manganese, lead, zinc, nitrate, sulphate, fluoride, phosphate, and pH, following the methods of APHA [22] and WHO [16]. The pH and temperature of the water samples were measured using a digital pH meter with a temperature probe. The TDS and EC of the samples were measured by a portable digital TDS-EC meter. The remaining physicochemical parameters were analyzed using Hach Model DR/2400 Portable Spectrophotometer.

#### 2.3.2. Bacteriological Analysis of Water Sources

Water samples collected in presterilized plastic bags were filtered using membrane filters with a spore size of 45 *μ*m diameter. The filters were placed in sterilized Petri dishes with absorbent pads flooded with Lauryl Sulphate Broth [16, 22] and then incubated at 37°C and 44°C for total coliform and faecal coliform, respectively. The filters were examined after 24 hours to assess bacterial growth. Based on the number of colonies of coliforms, the risk level of water source was assessed according to WHO guidelines [16], which were as follows: bacterial colonies <1, “very low risk”; 1–10, “low risk”; 11–100, “medium risk”; >100, “high risk” or “very high risk.”

### 2.4. Data Analysis

Data were analyzed using SPSS statistical software (version 20). Our results of physicochemical analysis and bacterial counts were compared with the standards set for drinking water quality [23, 24] and interpreted as acceptable or unacceptable. Mean separations between samples were computed using one-way ANOVA and DMRT post hoc tests. The parameters were correlated with each other to determine their relationship using Pearson's correlation. In all cases, significance was considered at a 95% confidence interval or *p* < 0.05 was considered statistically significant.

## 3. Results

### 3.1. Bacteriological Load of Rural Community Drinking Water Sources in Guto Gida District

All water samples were analyzed for total coliform (TC) and faecal coliform (FC) load. Both TC and FC were detected in all water samples from unprotected wells with counts ranging from 19 to 91.7 and 2.3 to 18.3 CFU/100 mL, respectively, with statistically significant variations among various sampling points (Table 1). The highest TC count was recorded in S2, whereas the least was in the water samples from S1. FC counts less than 10 CFU/100 mL were obtained in only 37.5% of water samples from unprotected wells. Similarly, both TC and FC were detected in all protected well water samples except D1, which was negative for FC (Table 1). Of all protected wells, the highest TC count was recorded in water samples from D2 followed by J1, with counts of 12.3 and 11.7 CFU/100 mL, respectively. Generally, FC counts less than 10 CFU/100 mL were noted in all protected wells without showing statistically significant variations (*p* > 0.05) among different sites. Both TC and FC counts of all water samples from protected wells were less than those of unprotected wells (Table 1).

All water samples from unprotected springs were positive for both TC and FC with counts ranging 16.3–59.7 and 2.7–20.7 CFU/100 mL, respectively (Table 1). In contrast, 37.5% of protected spring water samples were negative for both TC and FC (Table 1). Surprisingly, TC and FC counts less than 10 CFU/100 mL were counted in 100% protected spring water samples (Table 1). Both TC and FC counts of water samples from protected springs were lower than those of unprotected springs except K2 site, where 6.7 CFU/100 mL FC count was noted (Table 1).

Generally, overall mean TC counts of 51.4 and 6.6 CFU/100 mL were recorded for unprotected and protected well water sources. Likewise, mean FC counts of 11.5 and 1.5 CFU/100 mL were counted in water samples from protected and unprotected wells, respectively (Table 2). The mean TC and FC counts of water from protected wells were about tenfold lower than those from unprotected wells (Table 2). Statistically significant (*p* < 0.05) variations were observed among the mean values of TC and FC of both unprotected and protected wells (Table 2). The mean values of TC load recorded in unprotected spring and protected springs were 41.3 and 18.3 CFU/100 mL, whereas their mean FC counts were 10.4 and 1.84 CFU/100 mL, respectively (Table 2). Likewise, TC and FC counts of protected springs were significantly (*p* < 0.05) lower than those of unprotected springs (Table 2). Generally, TC and FC counts were in the order of unprotected wells > unprotected springs > protected springs > protected wells (Table 2).

According to WHO [16] risk level classification, 87.5% of drinking water sources considered in this study have TC counts of the category of medium risk, whereas 9.37% of them have fallen into the high risk category (Table 3). In case of FC counts, more than half (53.13%) of the water sources could be categorized as low risk (Table 3), while 25% of them were within the medium risk category. Compared to protected sources, more unprotected wells and unprotected springs were within the medium risk category based on TC counts. Based on FC counts, most protected wells and protected springs were within the low risk classification (Table 3).

### 3.2. Physical Characteristics of Rural Community Drinking Water Sources in Guto Gida District

The mean temperature values of water samples from different water sources were within the narrow range of 21.98–21.13°C, which were noted for unprotected wells and protected springs, respectively, without showing statistically significant (*p* > 0.5) variations (Tables 4–6). Of the total water samples, the highest temperature value of 24.62°C was recorded in the unprotected well of J2, whereas the lowest of 20.16°C was noted for protected springs at S1. Generally, unprotected water sources have shown higher temperature values than protected sources (Table 6).

The highest pH value of 7.05 was recorded for the water samples from the unprotected well of D1, whereas the lowest pH of 5.63 was recorded for protected springs of J1. In about 60% of the water samples, pH values < 6.5 were recorded. In most water samples from unprotected wells and protected springs, pH values < 6.5 were recorded, except D1 and D2 sites of unprotected wells and K2 of protected springs (Tables 4 and 5). The overall mean pH values of water samples from unprotected wells, unprotected springs, protected well, and protected springs were 6.28, 6.71, 6.33, and 6.12, respectively (Table 6). Generally, the pH values of unprotected springs are significantly higher than the pH values of other water sources (*p* < 0.05).

The highest EC value was recorded in the water sample from the unprotected well of J2 (549.6 *μ*S/cm), whereas the lowest value was recorded in unprotected spring water from D1 (225 *μ*S/cm) (Tables 4 and 5). Generally, water samples from protected sources had more EC values than those from unprotected sources (Table 6). Mean EC records from unprotected wells, unprotected sprig, protected well, and protected springs were 445.63, 305.54, 352.54, and 431.75 *μ*s/cm, respectively, with statistically significant variations (*p* < 0.05) among water samples from different sources and sites (Tables 4–6).

The turbidity of water samples from unprotected wells ranged from 2.09 to 8.01 NTU. The latter value is the highest of all sites noted for water samples from S2 (Tables 4–6). In contrast, turbidity values less than 5 NTU were noted in all sites of unimproved sources. The highest turbidity value was recorded in the water samples from the unprotected wells of S2 (8.01 NTU), whereas the lowest value (2.24 NTU) was noted in protected wells of the same sites. Unprotected water sources had more turbidity values than the protected ones (Table 6). In two samples from unprotected springs (D2 and J2) and one sample of unprotected well (D2), turbidity values above 5 NTU were recorded (Tables 4–6).

The highest and the lowest TDS values were recorded in water samples from the unprotected well of J2 (319.7 mg/L) and unprotected spring of J1 (156 mg/L), respectively (Tables 4 and 5). Fortunately, TDS values less than 500 mg/L were recorded in all cases (Tables 4 and 5). The TDS values of unprotected water sources were higher than those of protected water sources with significant variations among various water sources and sampling sites (*p* < 0.05) (Table 6). The mean color values of all water samples were in the range between 2.79 and 13.13 Pt.co. The highest water color value was recorded in D1 of the unprotected well (15.3 Pt.co), whereas the lowest was in J2 of the protected springs (7.33 Pt.co). Statistically significant variations were obtained among the water colors of water samples from different sources (*p* < 0.05).

### 3.3. Chemical Characteristics of Rural Community Drinking Water Sources in the Guto Gida District

Chemical characteristics are among the quality parameters for drinking water. Accordingly, rural community water sources were analyzed for selected chemical parameters to assess their suitability for drinking. Nitrate is a chemical parameter that commonly raises public health concerns. The concentration of nitrate obtained in water samples from unprotected wells ranged 31.9 mg/L to 11.7 mg/L (Table 7). The highest nitrate concentration was recorded in the unprotected wells of D1 (Table 7). Likewise, the concentration of nitrate in unprotected springs from various sites ranged from 32.46 mg/L to 5.66 mg/L (Tables 8 and 9). The highest value was noted in water samples from the D1 site (Tables 8 and 9). The concentration of nitrate noted in the protected wells ranged 14.4–6.7 mg/L (Table 10). These values are so far lower than the concentration of nitrate obtained in unprotected well and water sources (Tables 7 and 10). Similarly, nitrate concentrations lower than those of unprotected spring water samples were recorded in protected springs (Tables 8 and 9). Generally, a higher concentration of nitrate was noted in unprotected water sources than that of protected sources (Tables 7–10). Amazingly, all values conform to the standard set by both WHO and ES (<45 mg/L).

The mean sulphate concentrations of water sources were ranged 221–236 mg/L (Tables 7–10). The highest sulphate concentration was 286.67 mg/L noted in unprotected spring S2, whereas the lowest was 26 mg/L recorded for the protected springs of the same site (Tables 7 and 10). Significantly lower concentrations of sulphate ranged from 26.0 to 58.3 mg/L were obtained in protected wells (Table 10). Similarly, the concentrations of sulphate measured in unprotected springs are significantly higher than those of protected springs (Tables 8 and 9). Generally, the concentrations of sulphate obtained in unprotected sources have exceeded those of protected sources (Table 11). Generally, all values were lower than the maximum permissible level set by WHO for drinking water (400 mg/L), whereas S1 and S2 of unprotected wells exceed Ethiopian standards (250 mg/L). Mean phosphate concentrations of various sources ranged 0.73–0.69 mg/L without showing significant variations (*p* > 0.05) among sources and sites (Table 11). The highest phosphate concentration was obtained in unprotected spring J2, whereas the least (0.11 mg/L) was recorded from the unprotected spring of K1.

Fluoride is among the chemical parameters with a serious public health concern. The concentrations of fluoride recorded in water samples from three sites of unprotected wells were over the standard set by WHO (1.5 mg/L). These are S1, S2, and D2 with fluoride concentrations of 2.5, 2.0, and 1.87 mg/L, respectively (Tables 7–10). Similarly, water samples from three unprotected springs of various sites contained fluoride concentrations more than the maximum tolerable limit set by both WHO and ES (Tables 7–10). The sites are D1, K1, and J1 with fluoride concentrations of 3.81, 2.01, and 1.27 mg/L, respectively (Tables 7–10). Fluoride concentrations above permissible levels were detected in some protected wells and protected springs (Tables 7–10).

The concentrations of manganese obtained in water samples from unprotected wells of various sites ranged 0.01–0.9 mg/L (Table 7). The values noted in the majority of the sites are above the permissible level. Likewise, in most water samples from the protected spring, manganese concentrations above the tolerable level were recorded (Table 9). The concentrations of manganese in water samples from unprotected springs ranged 0.08–0.039 mg/L (Table 8). Of these, only two sites were found to be over the permissible level (Table 8). Generally, most samples of the unprotected springs and protected wells were within the permissible level (Tables 7 and 8).

The concentrations of iron recorded in water samples from unprotected wells of 4 sites were above the tolerable level (3 mg/L) (Table 7). Water samples from three sites of both protected wells and unprotected springs did not comply with WHO standards (Tables 8 and 10). In the majority of water samples from protected springs, iron concentrations above the permissible levels were noted (Table 9). Generally, most protected sources did not comply with both standards set by WHO and ES [17] (Tables 8 and 10).

The concentration of lead obtained in unprotected well water samples ranged 0.0–0.015 mg/L (Table 7), which conforms to the standard set by WHO (<0.05 mg/L). In the case of unprotected spring water sources, values above the permissible level were detected in two sites (J2 and K1) (Table 8). Similarly, the concentrations of lead obtained in J2 and K1 of protected spring and well water sources were above the permissible level set by WHO (Tables 8–10).

Zinc is a metal associated with deteriorated drinking water quality when it is available in concentrations beyond the permissible levels. The concentrations of zinc obtained in the drinking water samples from various sources ranged 2.2–0.4 mg/L (Table 8). The highest zinc concentration of 3.9 mg/L was recorded in protected spring of J2, whereas the low value of 0.5 mg/L was noted for protected spring D2 (Table 9). The concentrations of zinc obtained in all water sources and sites except S2 were more than 0.5 mg/L (Tables 7–10). Generally, higher concentrations of zinc were obtained in protected springs and protected wells (Table 11). Statistically significant variations were obtained among different water sources (*p* < 0.01).

## 4. Discussion

Our microbial load analysis of rural community drinking water sources showed that all water samples obtained from unprotected wells, unprotected springs, and protected wells had both TC and FC counts beyond both WHO and Ethiopian nationally acceptable levels for drinking water, i.e., 0 CFU/100 mL [16, 17]. A similar study carried out in the Jimma zone showed that well water sources were heavily polluted with TCs as high as 234 CFU/mL [13]. Another study conducted in the Bona district of Sidama zone reported that protected springs and wells had a high number of *E.coli* [12]. Likewise, a study conducted on the bacteriological and physicochemical quality of drinking water sources in the rural communities of Amahara regional state showed that most drinking water sources have coliform counts above the permissible level with high sanitary risk scores [23]. Other similar studies conducted in Jimma [18] and Gambella region [9] also revealed that drinking water contained coliform counts above both WHO [16] and Ethiopian drinking water standards [17] permissible levels, indicating that the water is heavily contaminated with faeces.

The results of the present study revealed that TC and FCs were detected in 100% and 87.5% water samples, respectively, indicating a high rate of contamination with animal and human faeces. Likewise, earlier studies showed that most water samples from unimproved sources in rural areas of Ethiopia were positive for both TC and FCs [14, 20, 23, 24]. The high counts of indicator bacteria in water sources could be attributed to poor construction and sanitation, existence of human activities, and grazing animals around the sources [23].

Temperature is one of the physicochemical parameters used to evaluate the quality of potable water. It affects many processes, including the rate of chemical reactions in the water body, reduction in solubility of gases, and amplification of taste and color of drinking water sources. The temperature values recorded in all cases were beyond the permissible limit [21]. The temperatures of the water sources were positively correlated with TDS (*r* = 0.661) and EC (*r* = 0.836) values of the water sources, which could be associated with increased solubility ions due to warm temperature. Similarly, earlier studies conducted on the quality of rural community drinking water sources indicated that the temperature values were >20°C in both improved and unprotected water sources [23], which might coincide with the climate conditions of the study areas.

The pH values of all water samples were found in the range of 5.63 to 7.05. However, only 34.4% of the water samples had pH values within the WHO recommended range, i.e., 6.5–8.5 [16]. In the majority of water sources, pH values less than 6.5 were recorded. Similarly, Berhanu and Hailu [12] reported that the pH values of 20% of the protected springs were <6.0, whereas the majority of protected springs and well water samples' pH values were in the permissible range. Low pH values recorded in protected water sources could be due to saturation with carbon dioxide [23] and acidic pH of soils of the study area. The pH values of water sources have shown statistically significant negative correlation with TDS, EC, iron, fluoride, manganese, and sulphate concentrations of the water sources (Table 12). In water sources with highly acidic pH, metals such as zinc, aluminum, and copper are released into the water since low pH is known to favor the solubility of ions associated with the high TDS value of the water [24].

In this study, about 12.5% of the total sample had turbidity levels above 5 NTU, which are beyond the acceptable standards of both Ethiopian and WHO [16, 17]. A previous study carried out at the Bona district of Sidama zone has revealed that 33% of protected springs and 17% of protected wells had turbidity values > 5 NTU [12]. Similarly, most rural drinking water sources in the Amhara region did not satisfy the turbidity value recommended by WHO [23]. The turbidity of water samples from various sources is positively correlated with bacterial load (*r* = 0.946), since higher turbidity is often associated with higher levels of suspended organic matter and microorganisms.

The electrical conductivity (EC) values of water sources in some sites were found to be above 500 *μ*mS/cm. Moreover, our results revealed that the EC values of rural community drinking water sources are positively correlated with nitrate (*r* = 0.540) and fluoride (*r* = 0.424) concentrations of the water. EC is directly related to the concentration of ions in water; the higher the ions, the higher the conductivity of the water sources. The highest EC values recorded in protected water sources could be due to the corrosion of metals that led to the accumulation of heavy metals, which might be due to acidic pH of the water sources. The lowest EC values were recorded in water samples obtained from unprotected springs and unprotected wells. Similar patterns were observed in protected wells and springs of the Jimma zone [24].

A TDS concentration in drinking water is associated with natural sources, sewage, urban runoff, industrial wastewater, and chemicals used in the water treatment process [24]. TDS affects the taste and odor of drinking water if present at levels above the WHO recommended level. The TDS included carbonate, bicarbonate, chloride, sulphate, phosphate, nitrate, calcium, magnesium, sodium, organic ions, and other ions [24]. The TDS values recorded in water samples from the study area are in agreement with the WHO value of 500 mg/L [21].

Our results have shown the presence of phosphate in water samples from the study area drinking water sources. Detection of phosphate in water sources indicates the contamination of the water sources by runoff from agricultural farms using inorganic fertilizers and urban sewage [12, 19]. In fact, all the water sources assessed in this study have a concentration of phosphate less than the maximum permissible level (5 mg/L) set by WHO [21]. The high phosphate concentrations in some of the water samples could be due to the presence of agricultural activities near the water sources. The concentration of nitrate in all water samples from study areas is within the permissible limit (50 mg/L) set by WHO [16]. The agricultural use of nitrates in chemical fertilizers could be a major source of water pollution. Prolonged exposure to nitrite and nitrate at levels above the maximum acceptable concentration could cause problems such as diuresis, increased starch deposits, and hemorrhaging of the spleen [1].

The concentration of lead in 30.2% of the water samples from various sources was above the Ethiopian maximum permissible level set for drinking water (>0.02 mg/L) [17]. Mebratu and Zerabruk [25] have also reported higher lead concentration in drinking water sources found in urban areas of Tigray, Ethiopia. Lead can also enter drinking water when service pipes that contain lead are corroded, especially where the water has high acidity or low mineral content that corrodes pipes and fixtures [8]. Zinc is another chemical parameter used to evaluate the quality of drinking water sources and all values recorded for various water sources were less than the permissible level. Although our results showed that zinc is not a water quality problem in the study area, marginally tolerable levels of zinc values were recorded in most sites in the case of both protected and unprotected wells. The concentration of zinc was also a bit higher in protected sources, which might be due to the dissolution of zinc from the pipes used in the construction of improved water sources. However, the overall results recorded in this study showed that almost all samples had zinc concentration within Ethiopian maximum permissible level (<5 mg/L) [17].

## 5. Conclusion

The bacteriological quality of most water samples analyzed in the current study did not meet the standards set for drinking water by both WHO and Ethiopian standards. The majority of the studied water sources could be classified as grossly polluted from a sanitary risk evaluation point of view and only very few of them had reasonable quality. Most of the water sources showed marginally tolerable quality with respect to color, EC, TDS, turbidity, nitrate, sulphate, and phosphate. However, protected sources have poor quality in relation to zinc, lead, iron, manganese, and pH with values much higher than the acceptable standards. Excessive lead, iron, and manganese concentrations recorded from some water samples could be related to pollution from corrosion of materials used for construction and agricultural sources.

Based on the results obtained from the analysis of water from the well and springs in the Guto Gida district, proper measures should be taken by the concerned bodies to ensure proper treatment of the waters to safeguard the health of the community. The measures should include continuous surveillance of water quality on a regular basis, public awareness creation, and the adoption of environmentally sound waste disposal methods to improve the quality of drinking water in the study area. Though improved sources could deliver safe water at the point of supply, the quality of drinking water could deteriorate during distribution and transport to households and then subsequent storage. Thus, further study on microbiological quality and water handling practices at the household level should be done at the study area to design community-based sustainable awareness creation programs and sound water supply chain management systems.

## Figures and Tables

**Table 1:** Total coliform and faecal coliform count of rural community drinking water sources in the Guto Gida district.

Sites	Unprotected wells		Protected wells		Unprotected springs		Protected springs	
	TC (CFU/100 mL)	FC (CFU/100 mL)	TC (CFU/100 mL)	FC (CFU/100 mL)	TC (CFU/100 mL)	FC (CFU/100 mL)	TC (CFU/100 mL)	FC (CFU/100 mL)
D1	75 ± 4.5^ab^	17 ± 5^ab^	3.3 ± 5.7^a^	0.0	59.67 ± 15.67^a^	20.67 ± 3.5^a^	0.0	0.0
D2	63.3 ± 30.8^ab^	17 ± 6.6^ab^	12.3 ± 12.5^a^	3.0 ± 3.0^a^	57.00 ± 10.53^a^	14.67 ± 5.69^ab^	1.67 ± 2.08^bc^	1.67 ± 2.08^b^
J1	41.7 ± 28.2^ab^	6.7 ± 7^bcd^	11.7 ± 4.9^a^	0.3 ± 0.6^a^	26.33 ± 18.8^ab^	6.67 ± 7.64^bc^	0.0	0.0
J2	50.3 ± 40.7^ab^	12.3 ± 7.1^abcd^	10 ± 2.7^a^	1.7 ± 1.5^a^	55.67 ± 37.56^a^	12 ± 8.54^abc^	2.67 ± 1.52^ab^	2.67 ± 1.52^b^
K1	50 ± 15.9^ab^	13.3 ± 3.1^abc^	5.3 ± 4.7^a^	1.7 ± 1.5^a^	38 ± 23.90^ab^	10.33 ± 9.07^abc^	0.0	0.0
K2	20.7 ± 8.1^b^	2.3 ± 2.1^d^	4.3 ± 7.5^a^	1.3 ± 2.3^a^	22.67 ± 7.57^ab^	4.33 ± 1.16^bc^	1.67 ± 1.52^a^	6.67 ± 2.52^a^
S1	19.0 ± 10.4^b^	4.7 ± 2.5^cd^	1.7 ± 1.5^a^	1.0 ± 1.7^a^	16.33 ± 8.39^b^	2.67 ± 1.16^c^	1.67 ± 1.16^bc^	1.67 ± 1.16^b^
S2	91.7 ± 45.2^a^	18.3 ± 8.7^a^	4.3 ± 4.0^a^	3.0 ± 2.7^a^	54.67 ± 9.29^a^	12.33 ± 1.52^abc^	2.0 ± 1.73^ab^	2.00 ± 1.73^b^

Data are average of triplicates; SE: standard error; numbers indicated by the same letter superscripts within the same column do not vary significantly by Duncan's multiple range test at *p* < 0.05; D1: Dalo site 1; D2: Dalo site 2; J1: Jato site 1; K1: Kumsa Moroda site 1; K2: Kumsa Moroda site 2; S1: Sorga site 1; S2: Sorga site 2; TC: total coliform; FC: faecal coliform.

**Table 2:** Overall mean total coliform and faecal coliform counts of rural community drinking water sources in the Guto Gida district.

Parameters	Water sources				
	Unprotected well	Unprotected spring	Protected well	Protected spring	*p* value
*n*	8	8	8	8	—
TC (CFU/100 mL) ± SE	51.4 ± 25.1^a^	41.3 ± 17.6^a^	6.62 ± 4.10^b^	18.25 ± 8.4^b^	<0.01
FC (CFU/100 mL) ± SE	11.46 ± 6.16^a^	10.4 ± 5.86^a^	1.50 ± 1.10^b^	1.84 ± 2.21^b^	<0.01

Data are average of 8 samples; SE: standard error; numbers indicated by the same letter superscripts within the same row do not vary significantly by Duncan's multiple range test at *p* < 0.05; TC: total coliform; FC: faecal coliform.

**Table 3:** Risk level classification of rural community's drinking water from unprotected and protected sources according to WHO [16].

Source	*n*	Total coliform				Faecal coliform			
		0	1–10	11–100	>100	0	1–10	11–100	>100
Unprotected well (%)	8	0	0	87.5	12.5	12.5	37.5	50	0
Protected well (%)	8	0	12.5	87.5	0	25	62.5	12.5	0
Unprotected spring (%)	8	0	0	100	0	12.5	50	37.5	0
Protected spring (%)	8	0	25	75	0	37.5	62.5	0	0

Bacterial colonies <1, “very low risk”; 1–10, “low risk”; 11–100, “medium risk”; >100, “high risk” or “very high risk,” *n*: number of water samples analyzed.

**Table 4:** Physical properties of rural community drinking water sources (unprotected well and protected wells) in the Guto Gida district.

Sampling sites	Unprotected wells						Protected wells					
	Temperature ± SE (°C)	pH ± SE	EC ± SE (*μ*S/cm)	Turbidity ± SE (NTU)	Color ± SE (Pt.Co)	TDS ± SE (mg/L)	Temperature ± SE (°C)	pH ± SE	EC ± SE (*μ*S/cm)	Turbidity ± SE (NTU)	Color ± SE (Pt.Co)	TDS ± SE (mg/L)
D1	20.7 ± 0.4^b^	6.7 ± 0.6^a^	426 ± 15^a^	4.8 ± 0.8^bc^	15.3 ± 1.5^a^	317 ± 120^a^	21.1 ± 1.6^ab^	6.2 ± 0.1^a^	416±8^a^	3.7 ± 0.5^abc^	12.3 ± 2.9^a^	252.7 ± 2.1^b^
D2	21.0 ± 3^ab^	6.5 ± 0.1^a^	420 ± 69^a^	5.9 ± 1.8^ab^	12.0 ± 2.0^b^	221 ± 102^a^	23.7 ± 0.7^a^	6.1 ± 0.4^a^	355 ± 124^a^	4.1 ± 1.3^abc^	10.0 ± 2.6^a^	214.3 ± 6.0^c^
J1	22.0 ± 0.5^ab^	6.0 ± 0.5^a^	463 ± 9 ^a^	2.9 ± 1.5^cd^	12.0 ± 1.0^b^	271 ± 24.6^a^	20.8 ± 1.1^b^	6.1 ± 0.1^a^	404 ± 46^a^	4.70 ± 0.2^ab^	10.0 ± 1.0^a^	27.7 ± 11.6^c^
J2	24.6 ± 1.0^a^	6.1 ± 1.2^a^	549 ± 66^a^	4.0 ± 1.6^cd^	12.3 ± 2.1^ab^	320 ± 25^a^	20.7 ± 2.1^b^	6.5 ± 0.4^a^	329 ± 99^a^	4.80 ± 0.2^a^	9.3 ± 2.1^a^	24.3 ± 4.9^c^
K1	21.6 ± 2.2^ab^	6.4 ± 0.5^a^	363 ± 211^a^	3.2 ± 1.1^cd^	13 ± 1.7^ab^	225 ± 119^a^	22.9 ± 1.4^ab^	6.3 ± 0.6^a^	358 ± 108^a^	4.14 ± 0.5^abc^	11.6 ± 3.1^a^	28.3 ± 4.5^c^
K2	21.1 ± 2.2^ab^	6.1 ± 0.3^a^	373 ± 112^a^	3.0 ± 0.8^cd^	13 ± 1.0^ab^	191 ± 88^a^	20.5 ± 1.0^b^	6.5 ± 0.3^a^	318 ± 19^a^	2.98 ± 1.0^cd^	13.3 ± 1.5^a^	187 ± 15.4^a^
S1	21.5 ± 1.4^ab^	6.4 ± 0.3^a^	454 ± 75^a^	2.1 ± 1.0^d^	13 ± 1.7^ab^	247 ± 71^a^	22.0 ± 1.8^ab^	6.5 ± 0.1^a^	298 ± 23^a^	3.4 ± 0.7^bcd^	9.0 ± 4.4^a^	191.3 ± 23.3^a^
S2	23.2 ± 2.5^ab^	6.1 ± 0.5^a^	515 ± 83^a^	8.0 ± 1.2^a^	14.3 ± 2.1^ab^	292 ± 32^a^	20.6 ± 0.8^b^	6.5 ± 0.6^a^	343 ± 147^a^	2.24 ± 0.4^d^	9.7 ± 2.5^a^	210.6 ± 8.3^a^

Data are average of triplicates; SE: standard error; numbers indicated by the same letter superscripts within the same column do not vary significantly by Duncan's multiple range test at *p* < 0.05; D1: Dalo site 1, D2: Dalo site 2; J1: Jato site 1; K1: Kumsa Moroda site 1; K2: Kumsa Moroda site 2; S1: Sorga site 1; S2: Sorga site 2; TDS: total dissolved solids; EC: electrical conductivity.

**Table 5:** Physical properties of rural community drinking water sources (unprotected springs and protected springs) in the Guto Gida district.

Sampling sites	Unprotected springs						Protected springs					
	Temperature	pH	EC	Turbidity	Color	TDS	Temperature	pH	EC	Turbidity	Color	TDS
D1	20.97 ± 0.40^a^	7.05 ± 0.47^a^	225.0 ± 87.9^c^	4.74 ± 0.20^ab^	14.0 ± 2.0^a^	179 ± 74.0^a^	20.9 ± 1.4^ab^	6.35 ± 0.4^abc^	360.7 ± 45.4^bc^	3.8 ± 0.4^abcd^	11 ± 2.65^a^	208 ± 20.66^ab^
D2	20.98 ± 2.91	6.13 ± .25^c^	406.0 ± 48.1^a^	5.01 ± .71^a^	12.3 ± 2.31^ab^	222.3 ± 48.0^a^	20.44 ± .77^ab^	6.32 ± .29^abc^	386.7 ± 109.2^bc^	4.90 ± .84^a^	10.33 ± 2.08^a^	232.3 ± 64.13^ab^
J1	21.21 ± 1.02^a^	6.60 ± .18^abc^	256.0 ± 65.4^bc^	2.86 ± 1.52^b^	11.67 ± 2.08^ab^	156 ± 44.8^a^	22.2 ± 0.91^ab^	5.63 ± .37^c^	540 ± 25.06^a^	4.41 ± 0.54^abc^	10.3 ± 2.08^a^	308.3 ± 36.8^a^
J2	23.91 ± 2.19^a^	6.90 ± 0.30^ab^	243.67 ± 95.8^c^	5.45 ± 1.47^a^	14.33 ± 0.58^a^	166 ± 37.47^a^	22.13 ± 0.24^a^	6.24 ± 0.21^abc^	452.3 ± 44.0 ^abc^	4.80 ± 0.18^ab^	7.67 ± 3.8^a^	252 ± 15.8^ab^
K1	21.76 ± 2.23^a^	6.30 ± 0.54^bc^	394.3 ± 46.3^ab^	3.73 ± 1.1^ab^	10.67 ± 0.58^b^	219.3 ± 40.6^a^	20.61 ± 0.87^ab^	5.60 ± 0.32^c^	488 ± 23.64^ab^	3.46 ± 1.14^bcd^	11.3 ± 3.06^a^	267.6 ± 21.4^ab^
K2	21.40 ± 1.89^a^	6.71 ± 0.19^abc^	297 ± 48.5^ab^	4.04 ± 0.79^b^	12.33 ± 1.52^ab^	190.3 ± 12.9^a^	21.1 ± 1.35^ab^	6.63 ± 0.08^a^	340.3 ± 37.3^c^	2.91 ± 1.09^d^	12.3 ± 4.51^a^	183.7 ± 16.86^b^
S1	20.89 ± 1.27^a^	6.96 ± 0.25^ab^	254.0 ± 93.8^bc^	4.56 ± 0.56^ab^	12.67 ± 1.5^ab^	157.3 ± 36.5^a^	20.16 ± 0.44^b^	6.44 ± 0.71^ab^	406.7 ± 117.9^abc^	3.27 ± 0.56^cd^	8.0 ± 1.00^a^	244.7 ± 80.9^ab^
S2	23.07 ± 2.30^a^	7.02 ± 0.59^ab^	368.3 ± 99.17^ab^	2.89 ± 1.33^b^	14.33 ± 1.16^a^	204 ± 71.08^a^	21.41 ± 1.0^ab^	5.72 ± 0.52^cd^	479.3 ± 114.2^abc^	2.40 ± 0.45^d^	7.33 ± 1.16^a^	288 ± 90.95^a^

Data are average of triplicates; SE: standard error; numbers indicated by the same letter superscripts within the same column do not vary significantly by Duncan's multiple range test at *p* < 0.05; D1: Dalo site 1; D2: Dalo site 2; J1: Jato site 1; K1: Kumsa Moroda site 1; K2: Kumsa Moroda site 2; S1: Sorga site 1; S2: Sorga site 2; TDS: total dissolved solids; EC: electrical conductivity.

**Table 6:** Overall mean values of physical characteristics of rural community drinking water samples from both unprotected and protected sources.

Physical	Water sample sources				
Parameters	Unprotected well	Unprotected spring	Protected well	Protected spring	*p* value

Temp (°C)	21.98 ± 1.32^a^	21.77 ± 1.12^a^	21.54 ± 1.2^a^	21.13 ± 0.76^a^	>0.05
pH	6.28 ± 0.25^b^	6.71 ± 0.35^a^	6.33 ± 0.2^b^	6.12 ± 0.40^b^	<0.01
EC (*μ*S/cm)	445.63 ± 64.42^a^	305.54 ± 73.1^b^	352.54 ± 40.5^b^	431.75 ± 69.32^a^	<0.01
Turbidity (NTU)	4.23 ± 1.94^a^	4.16 ± 0.95^a^	3.76 ± 0.87^a^	3.74 ± 0.90^a^	>0.05
Color (Pt. Co)	13.13 ± 1.17^a^	2.7.9 ± 1.33^a^	10.67 ± 1.57^b^	9.79 ± 1.88^b^	<0.01
TDS (mg/L)	260.58 ± 47.17^a^	186.79 ± 26.6^b^	209.54 ± 19.8^b^	248.08 ± 40.77^a^	<0.01

Data are average of triplicates; SE: standard error; numbers indicated by the same letter superscripts within the same row do not vary significantly by Duncan's multiple range test at *p* < 0.05, TDS: total dissolved solids; EC: electrical conductivity.

**Table 7:** Chemical characteristics of unprotected wells serving as water sources for the rural communities in the Guto Gida district.

Sampling sites	Iron (mg/L)	Fluoride (mg/L)	Manganese (mg/L)	Nitrate (mg/L)	Sulphate (mg/L)	Zinc (mg/L)	Lead (mg/L)	Phosphate (mg/L)
D1	0.2 ± 0.15^b^	0.8 ± 0.53^ab^	0.01 ± 0.01^c^	31.9 ± 6.3^c^	186.7 ± 27.7^bc^	1.9 ± 0.6^a^	0.03 ± 0.03^ab^	0.85 ± 0.43^a^
D2	0.1 ± 0.01^b^	0.9 ± 0.82^ab^	0.14 ± 0.16^bc^	25.8 ± 3.4^abc^	135.7 ± 33.6^c^	1.1 ± 0.9^a^	0.00 ± 0.00^b^	0.80 ± 0.18^a^
J1	0.4 ± 0.1^ab^	0.9 ± 0.5^ab^	0.82 ± 0.42^ab^	21.1 ± 1.2^bcd^	230.0 ± 50.5^ab^	0.8 ± 0.6^a^	0.00 ± 0.0^b^	0.80 ± 0.69^a^
J2	0.3 ± 0.3^ab^	1.0 ± 0.6a^ab^	0.90 ± 0.64^a^	29.4 ± 5.7^ab^	230.7 ± 54.4^ab^	0.8 ± 0.7^a^	0.04 ± 0.1^ab^	0.68 ± 0.43a
K1	0.4 ± 0.18^ab^	0.2 ± 0.12^b^	0.44 ± 0.46^abc^	12.2 ± 10.1^d^	231.0 ± 58.0^ab^	1.4 ± 1.0^a^	0.15 ± 0.1^a^	0.31 ± 0.02^a^
K2	0.3 ± 0.14^ab^	0.1 ± 0.09^b^	0.54 ± 0.27^abc^	11.7 ± 6.0^d^	197.3 ± 86.6^a^	0.7 ± 1.1^a^	0.11 ± 0.1^ab^	0.89 ± 0.63^a^
S1	0.1 ± 0.004^b^	0.1 ± 0.04^b^	0.76 ± 0.27^ab^	14.1 ± 4.7^d^	270.0 ± 19.7^ab^	2.0 ± 1.6^a^	0.01 ± 0.0^b^	0.86 ± 0.09^a^
S2	0.7 ± 0.5^a^	1.41 ± 1.21^a^	0.85 ± 0.41^ab^	17.8 ± 2.08^cd^	286.67 ± 10.0^a^	2.5 ± 1.1^a^	0.04 ± 0.1^ab^	0.65 ± 0.23^a^

Data are average of triplicates; SE: standard error; numbers indicated by the same letter superscripts within the same column do not vary significantly by Duncan's multiple range test at *p* < 0.05, D1: Dalo, J: Jato, K: Kumsa Moroda, S1: Sorga Site one, S2: Sorga Site two.

**Table 8:** Chemical characteristics of unprotected springs serving as water sources for the rural communities in the Guto Gida district.

Sampling site	Iron (mg/L)	Fluoride (mg/L)	Manganese (mg/L)	Nitrate (mg/L)	Sulphate (mg/L)	Zinc (mg/L)	Lead (mg/L)	Phosphate (mg/L)
D1	0.14 ± 0.23^a^	0.15 ± 0.23^a^	0.11 ± 0.08^b^	6.71 ± 0.28^b^	209.7 ± 13.7^a^	3.81 ± 1.21^a^	0.02 ± 0.02^a^	0.50 ± .65^ab^
D2	0.47 ± 0.34^a^	0.55 ± 0.31^a^	0.17 ± 0.21^b^	11.13 ± 1.93^ab^	167.7 ± 36.9^ab^	0.89 ± 0.66^b^	0.01 ± 0.01^a^	0.73 ± 0.47^ab^
J1	0.41 ± 0.38^a^	0.08 ± 0.05^a^	0.15 ± 0.04^b^	11.08 ± 2.90^ab^	107.0 ± 14.2^bcd^	1.62 ± 1.37^b^	0.02 ± 0.02^a^	0.89 ± 0.73^ab^
J2	0.22 ± 0.17^a^	0.29 ± 0.23^a^	0.04 ± 0.07^b^	9.36 ± 4.22^ab^	94.0 ± 36.7^d^	1.85 ± .56^ab^	0.15 ± 0.09^a^	1.25 ± .44^a^
K1	0.40 ± 0.42^a^	0.60 ± 0.44^a^	0.11 ± 0.11^b^	14.41 ± 6.23^a^	153.7 ± 65.9^abc^	2.02 ± 2.26^b^	0.07 ± 0.04^a^	0.11 ± 0.18^b^
K2	0.13 ± 0.03^a^	0.42 ± .15^a^	0.29 ± 0.25^b^	10.61 ± 1.74^ab^	125.0 ± 20.5^bcd^	1.10 ± .88^b^	0.04 ± 0.07^a^	0.58 ± 0.48^ab^
S1	0.10 ± 0.12^a^	0.30 ± 0.30^a^	0.18 ± 0.16^b^	7.03 ± 0.50^ab^	107.3 ± 47.9^bcd^	1.15 ± .099^b^	0.02 ± 0.01^a^	0.44 ± 0.34^ab^
S2	0.17 ± .24^a^	0.21 ± 0.20^a^	0.62 ± 0.19^a^	11.52 ± 6.96^ab^	67.7 ± 26.5^d^	1.07 ± 0.15^b^	0.02 ± 0.02^a^	0.99 ± 0.49^ab^

Data are average of triplicates; SE: standard error; numbers indicated by the same letter superscripts within the same column do not vary significantly by Duncan's multiple range test at *p* < 0.05; D1: Dalo; J: Jato; K: Kumsa Moroda; S1: Sorga site 1; S2: Sorga site 2; TDS: total dissolved solids; EC: electrical conductivity.

**Table 9:** Chemical characteristics of protected springs serving as water sources for the rural communities in the Guto Gida district.

Sampling site	Iron (mg/L)	Fluoride (mg/L)	Manganese (mg/L)	Nitrate (mg/L)	Sulphate (mg/L)	Zinc (mg/L)	Lead (mg/L)	Phosphate (mg/L)
D1	0.53 ± 0.17^abc^	0.50 ± 0.33^abc^	0.56 ± 0.60^ab^	8.42 ± 1.61^c^	45 ± 13.8^b^	3.40 ± 0.79^ab^	0.02 ± 0.02^b^	0.70 ± 1.12^a^
D2	0.40 ± 0.49^bc^	0.60 ± 0.54^abc^	0.47 ± 0.47^ab^	11.3 ± 4.46^abc^	55.7 ± 16.8^ab^	1.89 ± 0.48^bcd^	0.01 ± 0.02^b^	0.62 ± 0.44^a^
J1	0.86 ± 0.18^ab^	0.98 ± 0.29^ab^	0.38 ± 0.05^ab^	18.19 ± 5.35^abc^	84.3 ± 9.7^a^	1.27 ± 0.67^cd^	0.01 ± 0.01^b^	1.10 ± 0.86^a^
J2	0.36 ± 0.25^bc^	0.56 ± 0.23^abc^	0.38 ± 0.14^ab^	9.64 ± 1.59^bc^	63.7 ± 5.0^b^	3.9 ± 2.02^a^	0.11 ± 0.10^ab^	0.14 ± 0.12^a^
K1	0.98 ± 0.21^a^	1.14 ± 0.15^a^	0.99 ± 0.01^a^	16.9 ± 3.45^ab^	85 ± 12.8^a^	2.53 ± 0.61^abc^	0.18 ± 0.20^a^	0.71 ± 0.09^a^
K2	0.88 ± 0.40^ab^	0.13 ± 0.06^c^	0.16 ± 0.10^b^	8.60 ± 0.44^bc^	33.3 ± 2.5^b^	2.0 ± 0.20^bcd^	0.00 ± 0.01^b^	0.78 ± 0.21^a^
S1	0.25 ± 0.37^c^	0.34 ± 0.54^bc^	0.51 ± 0.47^ab^	12.0 ± 6.5^abc^	39 ± 29.5^b^	2.17 ± 0.91^bcd^	0.01 ± 0.01^b^	0.59 ± 0.46^a^
S2	0.16 ± 0.08^c^	0.81 ± 0.44^abc^	0.64 ± 0.39^ab^	15.3 ± 6.5^abc^	66.0 ± 26.0^ab^	0.50 ± 0.34^d^	0.00 ± 0.01^b^	1.11 ± 0.25^a^

Data are average of triplicates; SE: standard error; numbers indicated by the same letter superscripts within the same column do not vary significantly by Duncan's multiple range test at *p* < 0.05; D1: Dalo; J: Jato; K: Kumsa Moroda; S1: Sorga site 1; S2: Sorga site 2.

**Table 10:** Chemical characteristics of protected wells serving as water sources for the rural communities in Guto Gida district.

Sampling sites	Iron (mg/L)	Fluoride (mg/L)	Manganese (mg/L)	Nitrate (mg/L)	Sulphate (mg/L)	Zinc (mg/L)	Lead (mg/L)	Phosphate (mg/L)
D1	0.62 ± 0.44^ab^	0.89 ± 0.18^ab^	0.11 ± 0.08^b^	6.71 ± 0.28^b^	27.3 ± 5.5^b^	3.81 ± 1.21^a^	0.02 ± 0.02^a^	0.80 ± 1.01^a^
D2	0.35 ± 0.24^b^	0.67 ± 0.37^ab^	0.17 ± 0.21^b^	11.13 ± 1.93^ab^	38 ± 13.6^ab^	0.89 ± 0.66^b^	0.01 ± 0.01^a^	0.41 ± 0.15^b^
J1	0.42 ± 0.22^b^	1.09 ± 0.15^a^	0.15 ± 0.04^b^	11.08 ± 2.90^ab^	39 ± 16.9^ab^	1.62 ± 1.37^b^	0.02 ± 0.02^a^	0.43 ± 0.46^b^
J2	0.17 ± 0.25^b^	0.56 ± 0.21^abc^	0.04 ± 0.07^b^	9.36 ± 4.22^ab^	33 ± 20.1^ab^	1.85 ± 0.56^ab^	0.15 ± 0.09^a^	0.97 ± 0.50^a^
K1	0.09 ± 0.03^b^	1.08 ± 0.15^a^	0.11 ± 0.11^b^	14.41 ± 6.23^a^	58.3 ± 24^a^	2.02 ± 2.26^b^	0.07 ± 0.04^a^	0.47 ± 0.43^b^
K2	0.11 ± 0.05^b^	0.07 ± 0.06^c^	0.29 ± 0.25^b^	10.61 ± 1.74^ab^	34 ± 2.6^ab^	1.10 ± 0.88^b^	0.04 ± 0.07^a^	0.70 ± 0.28^a^
S1	1.14 ± 0.80^a^	0.36 ± 0.58^bc^	0.62 ± 0.19^a^	7.03 ± 0.50^ab^	33.3 ± 4.2^ab^	1.15 ± 0.099^b^	0.02 ± 0.01^a^	1.08 ± 0.12^a^
S2	1.17 ± 0.15^a^	0.97 ± 0.30^a^	0.18 ± 0.16^b^	11.52 ± 6.96^ab^	26.0 ± 15.1^b^	1.07 ± 0.15^b^	0.02 ± 0.02^a^	0.77 ± 0.61^a^

Data are average of triplicates; SE: standard error; numbers indicated by the same letter superscripts within the same column do not vary significantly by Duncan's multiple range test at *p* < 0.05, D1: Dalo, J: Jato, K: Kumsa Moroda, S1: Sorga Site one, S2: Sorga Site two.

**Table 11:** Overall mean values of chemical properties of rural communities' drinking water from unprotected and protected sources in the Guto Gida district.

Chemical parameters	Water sample sources				
	Unprotected well	Unprotected spring	Protected well	Protected spring	*p* value
Nitrate (mg/L)	20.5 ± 7.85^a^	14.63 ± 9.97^ab^	10.23 ± 2.51^b^	12.56 ± 3.81^b^	<0.05
Manganese (mg/L)	0.20 ± 0.15^b^	0.21 ± 0.11^b^	0.21 ± 0.18^b^	0.51 ± 0.24^a^	<0.01
Iron (mg/L)	0.32 ± 0.20^a^	0.26 ± 0.15^a^	0.51 ± 0.43^a^	0.55 ± 0.31^a^	>0.05
Sulphate (mg/L)	221 ± 47.82^a^	129.00 ± 45.62^a^	59.00 ± 19.47^b^	36.29 ± 10.1^b^	<0.01
Zinc (mg/L)	1.40 ± 0.66^a^	0.44 ± 0.16^b^	1.69 ± 0.95^a^	2.21 ± 1.09^a^	<0.01
Phosphate (mg/L)	0.73 ± 0.19^a^	0.72 ± 0.31^a^	0.71 ± 0.25^a^	0.69 ± 0.36^a^	<0.01
Fluoride (mg/L)	0.56 ± 0.34^ab^	0.32 ± 0.19^b^	0.71 ± 0.37^a^	0.63 ± 0.33^ab^	>0.05
Lead (mg/L)	0.005 ± 0.005^a^	0.002 ± 0.003^a^	0.007 ± 0.009^a^	0.004 ± 0.006^a^	>0.05

Data are the average of 8; SE: standard error; numbers indicated by the same letter superscripts within the same column do not vary significantly by Duncan's multiple range test at *p* < 0.05.

**Table 12:** Pearson's correlation coefficient showing relationship among physicochemical and bacteriological parameters.

Parameter	Color	Turbidity	Temperature	pH	EC	TDS	Iron	Fluoride	Manganese	Nitrate	Phosphate	Sulphate	Zinc	lead	TC	FC
Color	1															
Turbidity	0.09	1														
Temperature	0.09	0.03	1													
pH	0.12	−0.08	−.15	1												
EC	0.14	0.13	0.84^*∗∗*^	−0.28	1											
TDS	0.30	0.19	0.66^*∗∗*^	−0.30	0.78^*∗∗*^	1										
Iron	−0.06	0.42^*∗*^	0.033	−0.65^*∗∗*^	0.09	0.16	1									
Fluoride	−0.33	0.022	0.329	−0.711^*∗∗*^	0.424^*∗*^	0.253	0.598^*∗∗*^	1								
Manganese	0.105	0.38	0.21	−0.46^*∗*^	0.21	0.09	0.76^*∗∗*^	0.59^*∗∗*^	1							
Nitrate	0.16	0.20	0.34	0.14	0.54^*∗∗*^	0.57^*∗∗*^	−0.22	−0.14	−0.30	1						
Phosphate	−0.11	−0.04	0.18	−0.24	0.03	0.07	0.39	0.43^*∗*^	0.36	−0.35	1					
Sulphate	0.01	−0.05	−0.15	−0.48^*∗*^	−0.02	0.09	0.10	0.01	−0.01	0.08	0.16	1				
Zinc	0.19	0.42^*∗*^	−0.17	−0.12	−0.07	0.01	0.04	0.08	0.06	−0.14	0.34	0.17	1			
Lead	−0.16	−0.15	−0.01	0.32	−0.43^*∗*^	−0.30	−0.14	−0.13	0.01	−0.39	0.23	−0.40	0.06	1		
TC)	0.08	0.79^*∗∗*^	0.14	−0.17	0.16	0.42^*∗*^	0.40^*∗*^	0.06	0.18	0.24	0.15	0.00	0.33	−0.06	1	
FC	0.09	0.77^*∗∗*^	0.13	0.02	0.13	0.37	0.22	−0.08	0.01	0.31	0.01	−0.14	0.34	−0.02	0.95^*∗∗*^	1

^*∗*^Correlation is significant at the 0.05 level (2-tailed). ^*∗∗*^Correlation is significant at the 0.01 level (2-tailed).

## Data Availability

All the necessary data supporting this research article are included in the manuscript. Raw data supporting this study will be accessed from the corresponding author or first author on reasonable request.

## Abbreviations

APHA: American Public Health Association
ESA: Ethiopian Standard Authority
CSA: Central Statistical Agency
WHO: World Health Organization
FC: Faecal coliform
TC: Total coliform
CFU: Colony-forming units
EC: Electrical conductivity
TDS: Total dissolved solids
MoH: Ministry of Health
D1: Dalo site 1
D2: Dalo site 2
J1: Jato site 1
J2: Jato site 2
K1: Kumsa Moroda site 1
K2: Kumsa Moroda site 2
S1: Sorga site 1
S2: Sorga site 2.
